# Carbohydrate regulation response to cold during rhizome bud dormancy release in *Polygonatum kingianum*

**DOI:** 10.1186/s12870-022-03558-0

**Published:** 2022-04-01

**Authors:** Yue Wang, Tao Liu, Changjian Ma, Guoqing Li, Xinhong Wang, Jianghui Wang, Jin Chang, Cong Guan, Huimin Yao, Xuehui Dong

**Affiliations:** 1 Shandong Academy of Agricultural Sciences, Jinan, Shandong China; 2 Key Laboratory of East China Urban Agriculture, Ministry of Agriculture and Rural Affairs, Jinan, Shandong China; 3 Tai’an Academy of Agricultural Science, Taian, Shandong China; 4 Shan Dong Agriculture and Engineering University, Jinan, Shandong China; 5 Tai’an Academy of Forestry Sciences, Taian, Shandong China; 6 China Agricultural University, Beijing, China

**Keywords:** *P. kingianum*, Rhizome bud dormancy, Carbohydrate metabolism

## Abstract

**Background:**

The rhizome of *Polygonatum kingianum* Coll. et Hemsl (*P. kingianum*) is a crucial traditional Chinese medicine, but severe bud dormancy occurs during early rhizome development. Low temperature is a positive factor affecting dormancy release, whereas the variation in carbohydrates during dormancy release has not been investigated systematically. Therefore, the sugar content, related metabolic pathways and gene co-expression were analysed to elucidate the regulatory mechanism of carbohydrates during dormancy release in the *P. kingianum* rhizome bud.

**Results:**

During dormancy transition, starch and sucrose (Suc) exhibited opposing trends in the *P. kingianum* rhizome bud, representing a critical indicator of dormancy release. Galactose (Gal) and raffinose (Raf) were increased in content and synthesis. Glucose (Glc), cellulose (Cel), mannose (Man), arabinose (Ara), rhamnose (Rha) and stachyose (Sta) showed various changes, indicating their different roles in breaking rhizome bud dormancy in *P. kingianum*. At the beginning of dormancy release, Glc metabolism may be dominated by anaerobic oxidation (glycolysis followed by ethanol fermentation). After entering the S3 stage, the tricarboxylic acid cycle (TCA) and pentose phosphate pathway (PPP) were may be more active possibly. In the gene co-expression network comprising carbohydrates and hormones, *HYD1* was identified as a hub gene, and numerous interactions centred on *STS/SUS* were also observed, suggesting the essential role of brassinosteroids (BRs), Raf and Suc in the regulatory network.

**Conclusion:**

We revealed cold-responsive genes related to carbohydrate metabolism, suggesting regulatory mechanisms of sugar during dormancy release in the *P. kingianum* rhizome bud. Additionally, gene co-expression analysis revealed possible interactions between sugar and hormone signalling, providing new insight into the dormancy release mechanism in *P. kingianum* rhizome buds.

**Supplementary Information:**

The online version contains supplementary material available at 10.1186/s12870-022-03558-0.

## Introduction

*Polygonatum kingianum* (*P. kingianum*) is an essential traditional Chinese medicinal plant included in the *Pharmacopoeia of the People’s Republic of China* in 2020 [1]. Its medicinal and health care value has been accepted by an increasing number of people, resulting in increased plant collection and reduced wild resources over time. Currently, vegetative propagation is the main cultivation method for *P. kingianum*, but long-term asexuality easily causes variety degradation and is not conducive to variety breeding. *P. kingianum* can reproduce sexually through its seeds; however, severe bud dormancy occurs at the stages of early rhizome development (Fig. S1F) [2], limiting *P. kingianum* production.

Chilling treatment can considerably promote bud dormancy release, and several studies have demonstrated that carbohydrates are closely related to this process [3]. Starch and sucrose (Suc) showed opposite dynamic trends in content and anabolism during seasonal shift-related processes [4]; for example, starch degradation pathways were significantly enriched [5], but the monosaccharide content increased gradually in beans during dormancy break [6], and such metabolic changes were also observed in sweet potato [7] and ginger [8]. Interestingly, the reducing and nonreducing sugar contents also showed inverse changes during bud dormancy [9]. Additionally, applying glucose (Glc), Suc and maltose to roots could promote the germination of *Lepidopsis thunb* [10], with interesting changes in Glc metabolism during bud dormancy release. The proportion of pentose phosphate pathway (PPP) activity in respiration increased gradually after the release of peony bud dormancy induced by cold treatment, while the proportion of Embden-Meyerhof-Parnas (EMP) pathway activity decreased gradually [11]. Similarly, grape buds whose dormancy had been released by chemical treatment exhibited increased PPP activity [12, 13]. However, opposite results have also been reported; for example, the EMP pathway and tricarboxylic acid cycle (TCA) cycle activities were improved after dormancy break by cold treatment in tree peony and grape buds [14, 15], likely explaining the dormancy transition, while PPP activity decreased gradually with sprouting in apple [16] and in other plant buds [17, 18]. Carbohydrates do not play a separate role in responding to low temperature to regulate bud dormancy but interact with hormone-related molecules. For example, abscisic acid (ABA) synthesis mutants were less responsive to Glc or Suc [19]. Additionally, the indole-3-acetic acid (IAA) biosynthesis gene *ZmYUCCA* could be regulated by sugars [20], and gibberellin (GA) treatment influenced sucrose phosphate synthase (*SPS*)/sucrose synthase (*SUS*) expression and Suc accumulation [21]. The above results suggest that sugar molecules play an essential role in regulating bud dormancy [22].

*P. kingianum* is a crucial traditional Chinese medicine; however, rhizome bud dormancy hinders reproduction, and the mechanism of bud dormancy release promoted by low temperature remains unclear, particularly the carbohydrate regulation response to cold during bud dormancy release. Additionally, polysaccharides are the main medicinal component, and the polysaccharide biosynthetic pathway remains to be explored in *P. kingianum*. The current study investigated carbohydrate metabolism and cross-links with phytohormones responding to cold during bud dormancy release in *P. kingianum*. This study aimed to identify valuable genes associated with bud dormancy and provide a foundation for breeding excellent germplasms without bud dormancy. Additionally, our report will provide valuable clues to identify candidate genes involved in polysaccharide biosynthesis and facilitate future studies on the molecular mechanisms of polysaccharide biosynthesis.

## Materials and methods

### Plant materials

*P. kingianum* seeds were collected from Hongpo village, Deqin County, Yunnan Province, China (27°48′46.03″N, 99°47′55.12″E) and were identified by Professor Xuehui Dong, College of Agronomy and Biotechnology, China Agricultural University. We collected *P. kingianum* seeds from landowners after acquiring their permission, and no other specific permissions were required for collection. The field study was supported by the Yunnan Drug Regulatory Administration and did not involve endangered or protected species.

The *P. kingianum* seeds were cultivated using wet sand with diameters of 0.05–0.8 mm in a growth chamber in the dark at 25 °C for approximately 46 days until the rhizome buds elongated to approximately 3–6 mm (Fig. S1f), after which the buds began to enter the endodormant state. Well-developed primary rhizomes were placed at 4 °C for different durations (0 d, 15 d, 30 d, 45 d, 60 d, 75 d, 90 d and 105 d). In our previous study [2], we identified three dormancy stages of *P. kingianum* buds: S1 (endodormancy, the bud was subjected to cold treatment for 15 d and was in a quiescent state), S2 (the bud was subjected to cold treatment for 30 d, and dormancy was partially released) and S3 (ecodormancy, the bud was subjected to cold treatment for 90 d, and dormancy was fully released). After each cold treatment, the rhizome buds were picked off. Some rhizome buds were quickly frozen in liquid nitrogen and stored at -80 °C for sugar detection and RNA extraction, and the other samples were stored in formalin-acetic acid-alcohol until sectioning for periodic acid-Schiff (PAS) staining.

### Carbohydrate content detection

Tissue extraction was conducted according to the Gesch [23] method. The rhizome bud was ground to powder in liquid nitrogen. Approximately 250–300 mg of sample was extracted with 80% ethanol 3 times at 85 °C. The mixed extraction solution was stabilized at 12 ml and then was incubated overnight at 4 °C. The supernatant was blown dry with nitrogen, after which the precipitate was dissolved in 2 ml of deionized water and filtered into a high-performance liquid chromatography (HPLC) injection vial using a 0.45-mm semipermeable membrane. An Aminex HPX-87 N column and refractive index detector were used for HPLC analysis of Glc, Suc, mannose (Man), galactose (Gal), rhamnose (Rha), arabinose (Ara), cellulose (Cel), raffinose (Raf) and stachyose (Sta). The precipitate generated after ethanol extraction in the above process was dried at 60 °C for starch analysis. After drying, the precipitate was incubated with KOH (0.2 M, 1 ml) in boiling water for 30 min, and then acetic acid (1 M, 0.2 ml) was added. Next, the solution was incubated in acetate buffer containing starch glycosidase (pH 4.6). After centrifugation, the supernatant was collected and dried with nitrogen, and the precipitate was dissolved in 2 ml of deionized water.

### Section preparation and PAS staining

The polysaccharide content in the same part of the rhizome buds after cold treatment for 0 d, 15 d, 30 d, 45 d, 60 d, 75 d, 90 d and 105 d was determined according to the method reported by Xu [24]. Paraffin sections with a thickness of 8 µm were obtained and stained with Schiff reagent. After staining, the sections were observed and photographed by using an OLYMPUS CCD optical microscope. ImageJ software was used to randomly select 10 sites in each slice to analyse density statistics.

### Quantitative real-time PCR analysis

Total RNA from S1, S2, and S3 rhizome buds was extracted with TRIzol reagent (Invitrogen, Carlsbad, CA, USA). Two micrograms of total RNA was used to synthesize first-strand cDNA with a SuperScript™ IV Kit (Thermo Fisher Scientific), and then the cDNA was diluted to 100 μl for qRT–PCR amplification. *UBQ7* (forward primer: 5’-ACCCCTTGTAATACCAGTGAC-3’, reverse primer: 5’-AATAGCAGGTCGTTTCC-3’) served as the internal control gene [2]. The expression of *STS* (forward primer: 5’-GTCATCACCTGCTCTACCAA-3’, reverse primer: 5’-TGATGGCTTGACTAAGTCCCT-3’), *SIP1* (forward primer: 5’-ATAAGAAATTGCGTCTCGAA-3’, reverse primer: 5’-GATTCATGTGTACTTTCCGAT-3’), *SPS3* (forward primer: 5’-ACGCTCTTTAGGTTACCAG-3’*,* reverse primer: 5’-AAGAGTATGAATTTCCTCGAT-3’) and *SUS3* (5’-AATGTTCCAAGTAAGGCCAT-3’, 5’-GCATTCTCATATTTTGCGAGT-3’) was analysed. The primers were designed by using Primer 5.0 and Oligo 7. qRT–PCR was performed with an Applied Biosystems™ 7500 Fast system. The relative expression of each gene was calculated by using the 2^−ΔΔCt^ method [25].

### Analysis of carbohydrate metabolic pathways

The transcriptomic data that were previously reported by our team (NCBI: SRA accession No. SRP149787) for S1, S2 and S3 rhizome buds were used for the current analysis. The upregulated and downregulated genes in both S2 vs. S1 and S3 vs. S2 were analysed by using Ingenuity Pathway Analysis and MapMan software, respectively.

## Results

### Changes in the carbohydrate content in *P. kingianum* buds

**Fig. 1:**
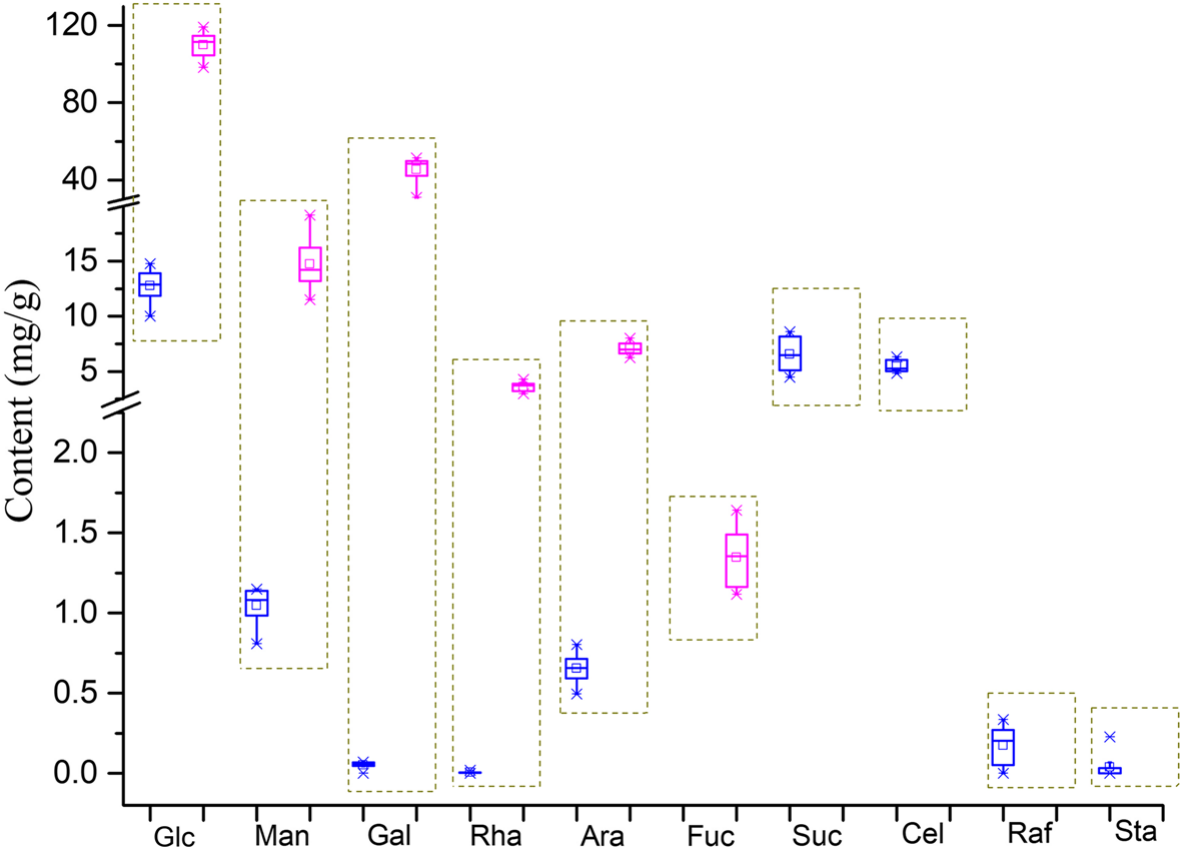
Distribution of the levels of the sugars in *P. kingianum* rhizome buds during dormancy release. Blue boxes represent free sugars, and pink boxes represent monosaccharide components of polysaccharides

The distribution of the free sugar content in all *P. kingianum* rhizome buds is shown in Fig. 1. The Glc content was the highest, with values of approximately 10–15 mg/g. The Suc and Cel levels ranged from 4 to 9 mg/g, and the Man and Ara levels ranged from 0.6 to 1.2 mg/g. The levels of Gal, Rha, Raf and Sta were the lowest and were all below 0.4 mg/g. The above results showed that Glc, Suc and Cel were the main free sugars in the *P. kingianum* rhizome buds. In contrast to free sugars, the monosaccharides derived from polysaccharides in the *P. kingianum* rhizome buds were Glc, Gal, Man, Ara, Rha and fucose (Fuc), listed here in the order of decreasing levels (Fig. 1).

**Fig. 2:**
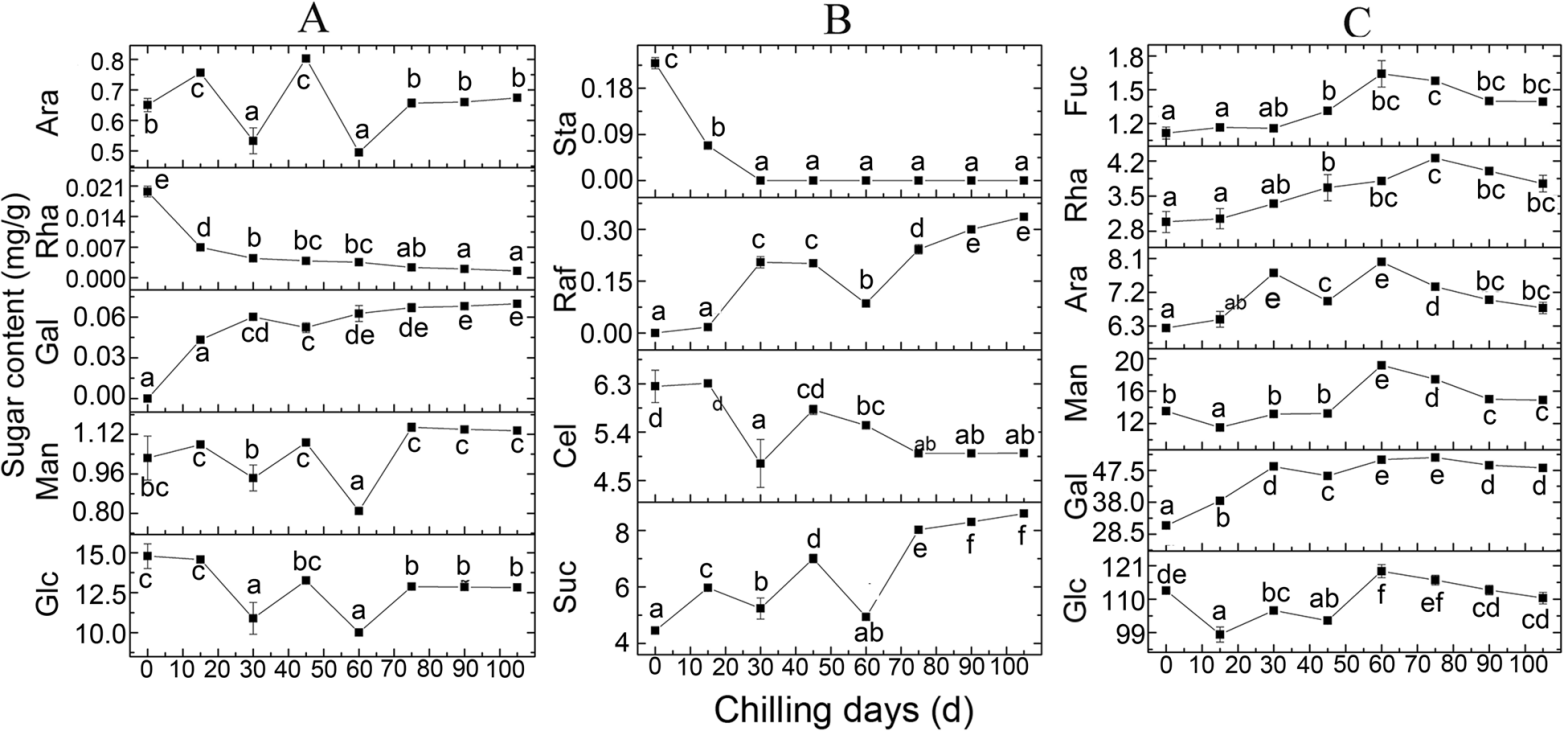
Dynamic changes in the sugar levels in *P. kingianum* rhizome buds during cold treatment. Free sugars are shown in (**A**) and (**B**), and monosaccharides derived from polysaccharides are shown in (**C**). Vertical bars indicate the standard error. Columns annotated by the same letter are not obviously different (*P* < 0.05)

During the whole chilling process, the Suc, Gal and Raf levels increased significantly (Fig. 2A and B). However, the levels of Rha, Sta and Cel were decreased and Sta decreased to 0 mg/g at later stages of cold treatment. The qRT–PCR results (Fig. S2) were not strictly consistent with the Suc and Sta levels, suggesting regulation at the level of protein translation. Other sugars, such as Ara, Man and Glc, showed no significant changes.

**Fig. 3:**
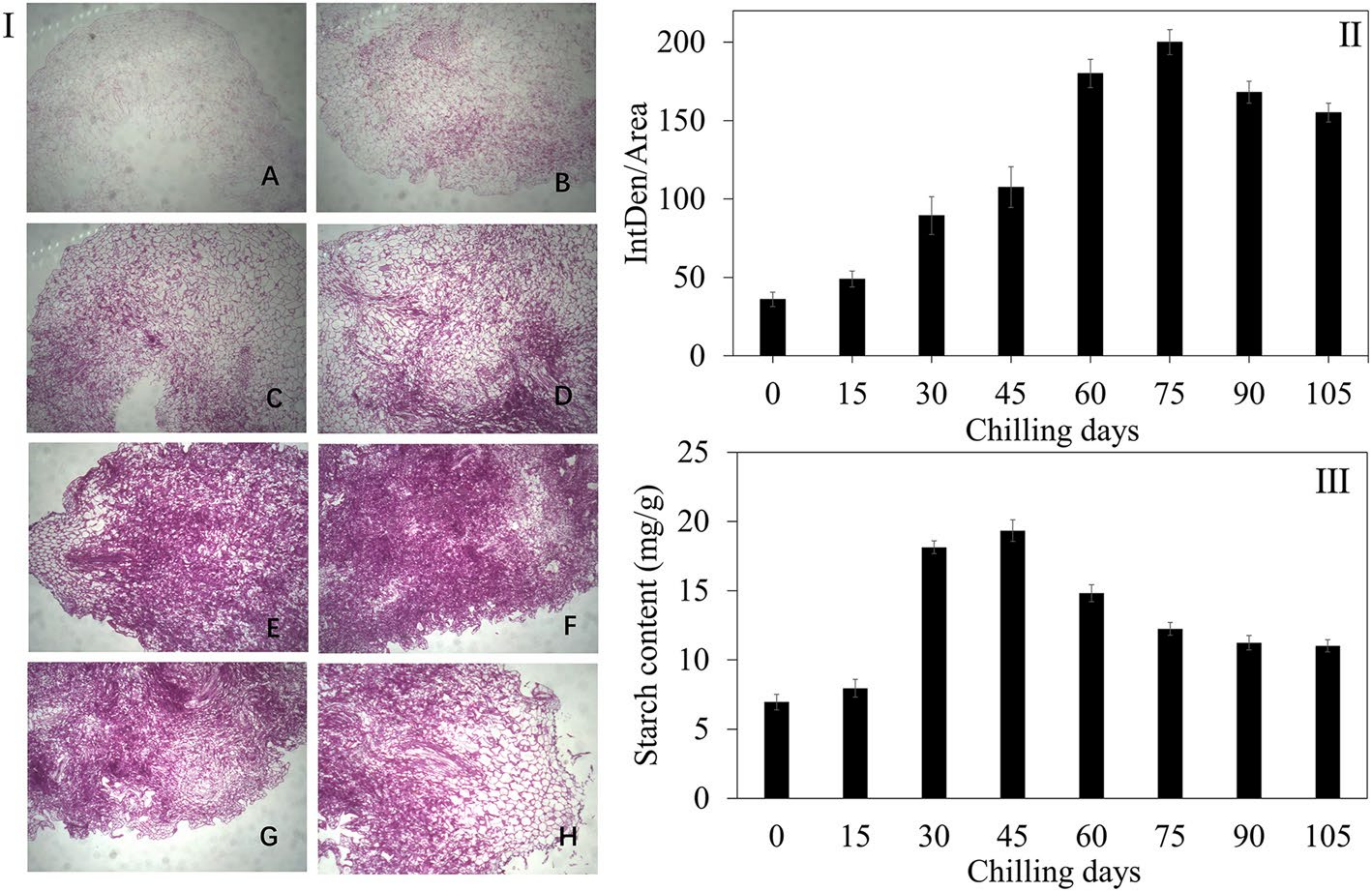
Dynamic changes in the polysaccharide levels in *P. kingianum* rhizome buds during cold treatment. (I) PAS staining of a *P. kingianum* rhizome bud section. The stained sections in A-H represent rhizome buds chilled for 0 d, 15 d, 30 d, 45 d, 60 d, 75 d, 90 d and 105 d, respectively. (II) Statistical analysis results for the staining density for PAS sections. (III) Dynamic change in the starch content

The PAS staining and densitometric analysis results (Fig. 3) showed that during 0–75 d of chilling treatment, the stain density gradually increased and peaked at 75 d, followed by a slight decrease, suggesting that the polysaccharide content increased significantly during 0–75 d and then decreased at later stages of cold treatment. Interestingly, all 6 monosaccharides showed increased followed by decreased levels as the number of days of cold treatment increased, and the peak value occurred at 60–75 d. This result was consistent with the PAS staining result. Additionally, the starch content was similar to that of polysaccharides, but the peak value occurred at 30–45 d.

### Analysis of the starch and sucrose metabolism pathway

**Fig. 4:**
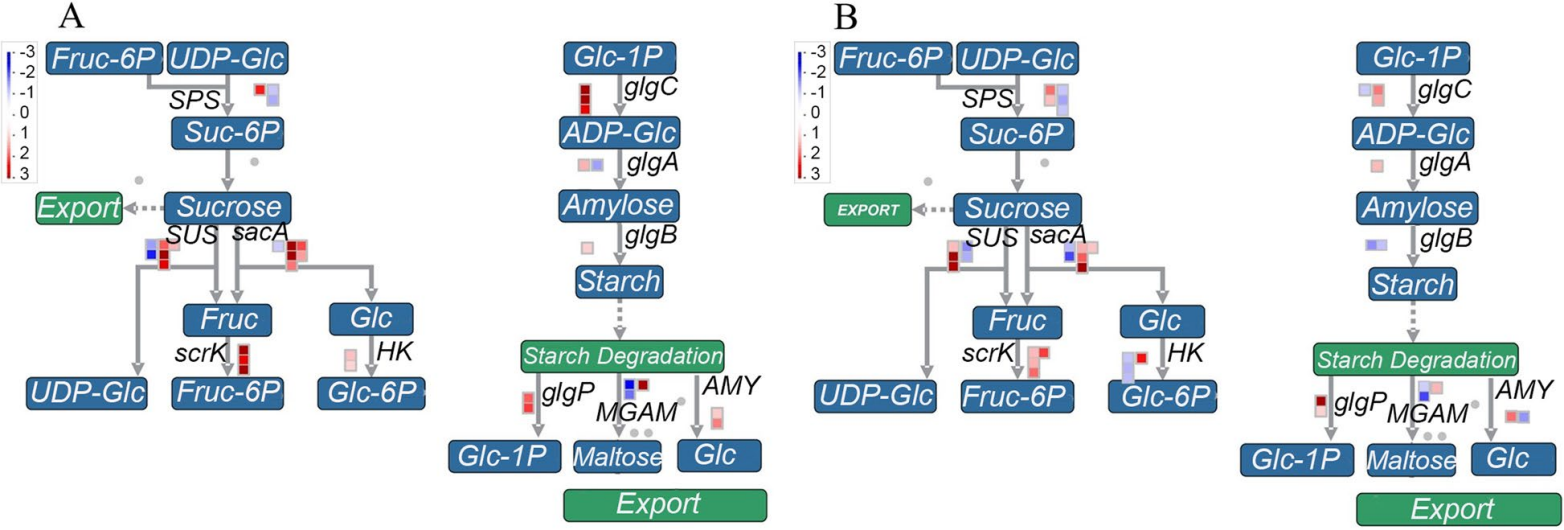
Analysis of starch and sucrose metabolism pathways during dormancy release in *P. kingianum* rhizome buds. Comparisons of S2 vs. S1 and S3 vs. S2 are shown in (**A**) and (**B**), respectively. Genes marked with red were upregulated, and genes marked with blue were downregulated

*SPS* catalyses the conversion of uridine diphosphate glucose (UDPG) to sucrose 6-phosphate (Suc-6P), which is the key enzyme for Suc synthesis. *SUS* is responsible for catalysing the conversion of Suc to UDPG and fructose (Fru). Fructofuranosidase (*sacA*) promotes the hydrolysis of Suc to Fru and Glc. The last step is the hydrolysis of Fru to Fru-6P by fructokinase (*scrK*). The expression levels of these genes mentioned above are shown in Fig. 4 and Table S1. *SPS* exhibited constitutive expression in both S2 vs. S1 and S3 vs. S2, and a difference may not exist in Suc synthesis during dormancy release. *SUS* and *sacA* showed increased expression in the comparison of S2 vs. S1 (the log_2_FC values of the 6 *SUS* transcripts were 1.93, 3.29, 2.59, 1.30, -1.49, and -2.29, and the log_2_FC values of the 6 *sacA* transcripts were 4.1, 1.77, 2.05, 1.48, 3.84, and -1.08) and S3 vs. S2 (the log_2_FC values of the 5 *SUS* transcripts were 1.23, 3.59, 2.94, -1.63, and -1.35, and the log_2_FC values of the 6 *sacA* transcripts were 1.35, 1.93, 4.28, 1.15, -1.26, and -2.05). Additionally, all the *scrK* transcripts were upregulated in S2 vs. S1 and S3 vs. S2. The above results indicated that a significant increase in Suc catabolism occurred during the process of dormancy release induced by cold temperature. This result was not consistent with the increased Suc content with the extension of chilling duration.

Adenosine diphosphate glucose (ADPG) provides glucosyl for starch synthesis; therefore, ADPG synthesis is a crucial step for starch synthesis, and glucose-1-phosphate adenylyltransferase (*glgC*) is responsible for this process. Starch synthase (*glgA*) catalyses the conversion of ADPG to amylose, which is then converted to starch by 1,4-alpha-glucan branching enzyme (*glgB*). Three key enzymes are involved in the catabolism of starch—glycogen phosphorylase (*glgP*), maltase-glucoamylase (*MGAM*) and beta-amylase (*AMY*). In the S2 vs. S1 group (Fig. 4A), the log_2_FC values of synthesis-related transcripts were 3.87, 3.32, 2.58, 1.31, -1.47 and 1.01, and the log_2_FC values of catabolism-related transcripts were 1.90, 2.12, 3.05, -2.48, -1.81, 1.09 and 1.71. Thus, in the early stage of dormancy release, both the synthesis and degradation of starch increased, but the increase in synthesis was greater than that of degradation, likely explaining the increase in the starch content during this period. In the S3 vs. S2 group, no large changes in the log_2_FC values of starch synthesis-related transcripts were observed (Fig. 4B). However, the degradation activity increased significantly, likely decreasing the starch content in the S3 stage (Fig. 3).

### Analysis of the galactose and glucose metabolism pathways

**Fig. 5:**
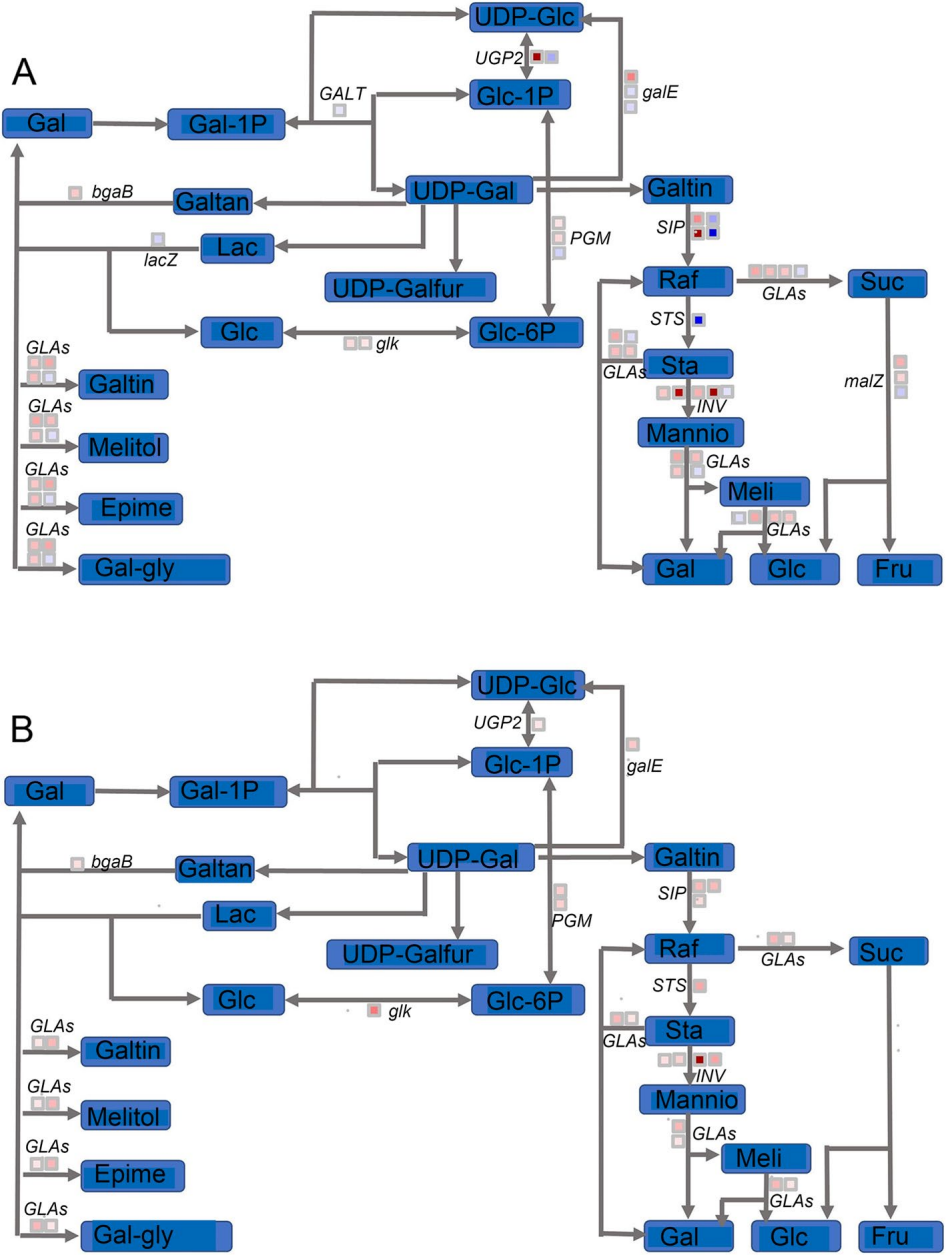
Analysis of the galactose metabolism pathway during dormancy release in the *P. kingianum* rhizome bud. Comparisons of S2 vs. S1 and S3 vs. S2 are shown in (**A**) and (**B**), respectively. Genes marked with red were upregulated, and genes marked with blue were downregulated. Galtan: Galactan; Lac: Lactose; UDP-Galfur: UDP-Galactofuranose; Galtin: Galactinol; Melitol: Melibiitol; Epime: Epimelibiose; Gal-gly: Galactosyl-glycerol; Mannio: Manniotnose; Meli: Meliose

Galactosidase (*GLA*) hydrolyses several galactoside glycans to Gal, and this reaction is a critical source of Gal. UDPG–hexose-1-phosphate uridylyltransferase (*GALT*) promotes the interconversion between galactose-1-phosphate (Gal-1-P) + UDPG and UDP-galactose (UDP-Gal) + glucose-1-phosphate (Glc-1-P), which is a key step in Gal metabolism. Inositol galactoside can be decomposed to Raf by raffinose synthase, and this reaction is an important source of Raf. Under the action of stachyose synthase (*STS*), Raf is converted to Sta and then to manninotriose. Finally, Gal can be produced again from manninotriose and melibiose. In the S2 vs. S1 group (Fig. 5), *GLAs* were upregulated, possibly increasing the Gal content in the S2 rhizome bud (Fig. 2A). Both upregulated and downregulated genes related to raffinose synthase (*SIPs* and *DIN10*) were observed, but the upregulation was stronger than the downregulation (Table S1). *STS* exhibited decreased expression, but *sacA* was upregulated. The above results showed that the synthesis of Raf increased, and the synthesis of Sta decreased, but the catabolism of Sta increased; therefore, the Raf content increased, but the Sta content decreased (Fig. 2B). Comparison of S3 vs. S2 revealed that the genes related to Gal metabolism were only enriched in upregulated pathways, particularly *GLA* and raffinose synthase genes, leading to increased Gal and Raf levels. Although stachyose synthetase-related genes were significantly upregulated, *sacA* was also significantly upregulated, likely leading to a balance between the synthesis and metabolism of Sta at this stage; therefore, the Sta content did not change significantly.

Three primary pathways of Glc catabolism exist: the EMP pathway, TCA cycle and PPP. The EMP pathway was only enriched in the upregulated differentially expressed gene (DEG) group for S2 vs. S1 but not S3 vs. S2 (Fig. S3). These genes included phosphoglucomutase (*PGM*), phosphohexose isomerase (*PHI*), aldolase (*ALD*), glyceraldehyde-3P-dehydrogenase, phosphoglycerate kinase (*PGK*), phosphoglycerate mutase (*PGAM*), enolase (*NSE*), pyruvate decarboxylase and acetaldehyde dehydrogenase (*ALDH*). In contrast to the EMP pathway, both the TCA cycle and PPP were only enriched in the upregulated DEG group for S3 vs. S2 but not for S2 vs. S1 (Table S1). This result showed that when dormancy release began (from S1 to S2), anaerobic oxidation in the glycolysis pathway may be the main way of Glc metabolism (with increased expression of pyruvate decarboxylase and *ALDH*). During the transition from S2 to S3, aerobic oxidation of glycolysis possibly became the main pathway (genes related to TCA were upregulated significantly), while the activity of PPP also increased at this stage.

### Gene co-expression analysis between carbohydrate and hormone metabolism

To examine possible interactions between carbohydrates and hormones during bud dormancy release in *P. kingianum*, 795 DEGs, including 518 DEGs related to carbohydrate metabolism that are mentioned above and 277 DEGs related to hormone metabolism that were reported in our previous study [2], were subjected to co-expression analysis. To ensure strong interactions among the genes, 673 pairs of interactions with correlation coefficients greater than 0.97 between 221 carbohydrate-related genes and 159 hormone-related genes (Table S2) were used to construct the co-expression network.

**Fig. 6:**
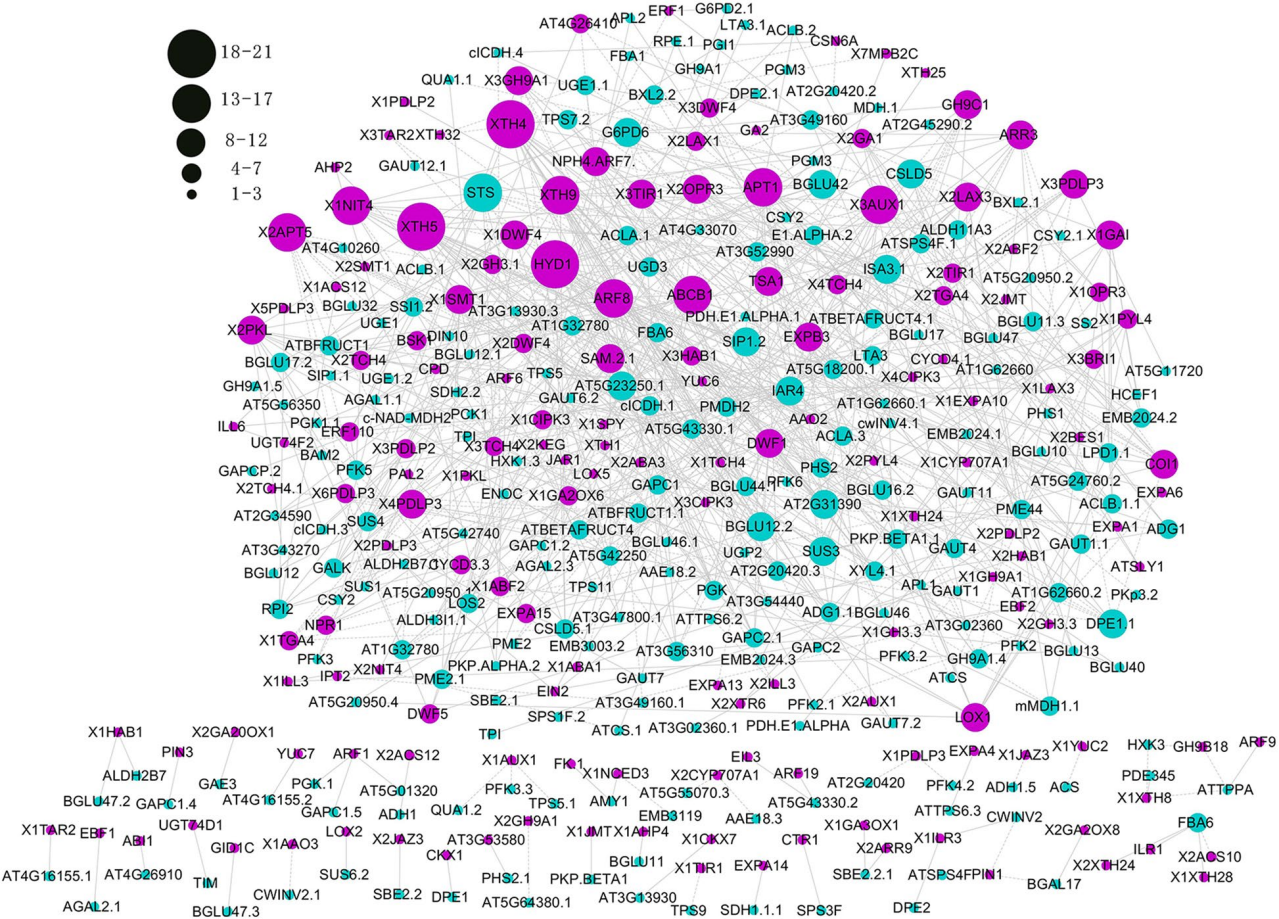
Gene co-expression network characterizing the interaction between carbohydrates and hormones. Circular nodes represent genes, and node size represents the degree of interaction of each gene. The degree values represented by different node sizes are marked in the upper left corner. The greater the degree or the more edges of a gene are available, the more edges are linked to the gene, and the more pivotal is the role it may play. Blue nodes represent genes related to carbohydrate metabolism, and red nodes represent genes related to hormone metabolism

In the network (Fig. 6), 501 pairs of interactions showed positive relationships, and 174 pairs of interactions exhibited negative relationships. All the genes were sorted into 5 groups by K-means analysis based on their degree of interaction. *HYD1* had the highest degree of interaction and was identified as a hub gene. *HYD1* is responsible for brassinosteroid (BR) synthesis, and 21 genes interacted with *HYD1*, including *AGAL1*, *GALK*, *ACLB*, *GAPC2*, *PGK*, *CSLD5*, *ACLA*, *BGLU*, *ACLA*, *ADG1*, *PME2*, and *ACLB*. These genes are involved in Gal metabolism, starch synthesis and glycolysis. *HYD1*, *XTH4*, *XTH5*, *ARF8*, *APT1*, *XTH9*, *APT5*, *ABCB1* and *AUX1* were the top 9 genes in terms of the degree of interaction and were all related to hormone metabolism. *STS*, which encodes stachyose synthase and is crucial for Sta synthesis, was the gene with the highest degree of interaction among the genes related to carbohydrate metabolism. Thirteen genes were shown to interact with *STS,* including *DWF4*, *BRI1*, *NIT4*, *TSA1*, *ILL6*, *ILL3*, *TIR1*, *ARR3* and *ERF110*. These genes are involved in the metabolism or signal transduction of BR, IAA, cytokinins and ethylene.

## Discussion

### Carbohydrates are crucial signalling molecules for bud dormancy

The present study showed that at the beginning of dormancy release, the starch synthesis activity increased and peaked at 30–45 d, leading to an increased starch content (Figs. 3 and 4). Subsequently, the starch content decreased continually, and the lowest value was reached during the S3 stage. However, in contrast to our study, the starch concentration decreased with increasing chill accumulation in sweet cherry buds [4, 26]. This difference may be due to the materials used in this study being the primary *P. kingianum* rhizome, which lacks nutrients; therefore, nutrient accumulation is necessary for life activities. Additionally, the seed and primary rhizome were still not separated at this stage, and the nutrients inside the seed could be transported to the primary rhizome through cotyledon coupling, causing the accumulation of nutrients. In fact, the starch content was reported to peak in the first 25 d of cold treatment in sweet cherry varieties “BING” and “SWEETHEART”, a finding that is consistent with ours [26].

Previous studies have shown an inverse relationship between the Suc and starch levels during dormancy release, which lasted until the bud entered ecodormancy [4], and this transformation was a crucial change during bud dormancy. In the current study, the Suc content increased continually during the whole cold treatment. Furthermore, the starch content showed a decreasing trend after 45 d of treatment. Therefore, this dynamic change also followed the “inverse relationship” rule. Ley [10, 27] proposed that low-molecular-weight carbohydrates (such as Suc, Glc and maltose) can promote bud dormancy release in *S. polyrrhiza,* and this promotion may be caused by osmotic effects; however, mannitol did not have this effect. In our study, the Suc, Raf and Gal levels increased significantly with bud dormancy release in *P. kingianum*.

In the *P. kingianum* rhizome bud, reducing sugars include Cel, Rha, Ara, Glc and Gal, and non-reducing sugars include Raf, Suc, Sta and Man. The levels of reducing and non-reducing sugars showed opposite trends during yam dormancy, with increased reducing sugar levels and decreased non-reducing sugar levels [9]. This previous conclusion is not consistent with our results (Fig. 2).

During the 0–105 d cold treatment period, the levels of all the monosaccharides derived from polysaccharides peaked at 60–75 d, a finding that is consistent with the PAS staining results. Polysaccharides are the main effective components in the *P. kingianum* rhizome and directly affect the medicinal value; therefore, determining the optimal cold treatment duration is critical. *P. kingianum* polysaccharides comprise 6 monosaccharides—Glc, Gal, Man, Ara, Rha and Fuc (Fig. 1). By contrast, in addition to the above monosaccharides, xylose was also reported as a polysaccharide component in *P. kingianum* from Wenshan. However, interestingly, Gal is not associated with *Polygonatum cyrtonema* polysaccharides, whereas only 4 monosaccharides are included in *Polygonatum sibiricum* [28]. Thus, significant differences were found in saccharides in *Polygonatum* among different species or geographical distributions.

### Changes in the glucose and nucleotide sugar metabolism pathways in dormancy release

Phosphofructokinase and pyruvate kinase are the essential enzymes to regulate the glycolysis rate, and a large amount of ATP is produced during the two steps. However, no significant changes related to these enzymes were observed in our study. Thus, the requirement for ATP might be fairly low at the beginning of bud dormancy release in *P. kingianum*. When the bud entered the S3 stage, the expression of genes involved in the TCA cycle, such as those encoding pyruvate decarboxylase, isolimonate dehydrogenase and α-ketoglutarate dehydrogenase, was upregulated. The TCA cycle can provide a large amount of ATP; thus, the demand for energy may increase substantially after endodormancy release in the *P. kingianum* rhizome bud. Besides, we monitored that several genes involved in EMP were upregulated only in S2 VS S1 group, however, both the TCA cycle and PPP were only enriched in the upregulated DEG group for S3 vs S2 but not for S2 vs S1, therefore, we speculated that EMP may be the main way of Glc metabolism at the beginning of dormancy release, in contrast, TCA and PPP were possibly more active after entering S3 stage. Actually, more physiological experiments were needed to verify this speculation, such as total respiration rate respiration ratio of EMP, TCA and PPP under different chilling treatments [13, 14], and we will continue to refine this point in future studies.

Nucleotide sugars are crucial precursors for polysaccharide synthesis; therefore, we also analysed the nucleotide sugar pathway. This pathway was enriched in the downregulated DEG group for S2 vs. S1 (Fig. S4) and the upregulated DEG group for S3 vs. S2. (Fig. S5). UDPG is the donor of several polysaccharides and the most widely studied nucleotide sugar [29]. Additionally, it is a crucial signal connecting carbohydrate metabolism with other metabolic pathways. For example, UDPG accumulation induces the utilization of sugars for lipid synthesis [30]. Previous studies have also shown that UDPG is a critical component of monosaccharides in *P. kingianum* [28]. Our study showed that genes responsible for catalysing the interconversion between Glc and UDPG as well as between UDPG and UDP-Gal/UDP-Rha/UDP-SQ/UDP-GlcA exhibited upregulated expression in S3 vs. S2 (Fig. S5). Furthermore, genes related to GDP-Man and GDP-Fuc were significantly downregulated in S2 vs. S1 but upregulated in S3 vs. S2. Man and Fuc are closely related to cold resistance [31], and our study indicated that they are also essential for chilling-induced dormancy release in *P. kingianum* buds. No significant changes were observed in the metabolic pathway of CDP-sugar. Additionally, Fru, Gal and Man are crucial energy substances that can be converted to phosphate-hexose and then enter the glycolysis pathway to provide energy. Hexokinase, *scrK* and *MPI*, which catalyse this process, showed a significant increase in S3 vs. S2 (Fig. S5), indicating that the entry of Fru and Man into glycolysis was enhanced following completion of dormancy release in *P. kingianum*.

### Regulatory mechanism of raffinose metabolism in rhizome bud dormancy release

*GLAs* promote the hydrolysis of several glycosides, which are the main components of the cell wall, to Gal. In our study, the Gal content increased obviously and peaked at the S3 stage (Fig. 2). This phenomenon reflected the active degradation of polysaccharides in the cell wall during this period. *GLAs* accelerate cell wall degradation and allow nutrients to flow out of the cell, promoting cell elongation and expansion. Additionally, the degradation products can also enter other metabolic pathways. Previous studies have shown that *GLA* expression increases during seed germination, which promotes endosperm weakening, facilitating the emergence of radicles through the seed coat [32]. A similar GLA-promoting mechanism may occur during dormancy release in *P. kingianum*.

Raf is a crucial nonstructural carbohydrate in plants [33] and plays an important role in cold stress, osmotic adjustment, antioxidant activity and energy storage [34]. Previous studies have shown that Raf accumulates during the seed germination process [35]. In the current study, the Raf content in rhizome buds increased significantly with dormancy release and peaked after endodormancy was entirely released, indicating that Raf plays critical roles in dormancy release induced by cold treatment. Overexpression of *OsGolS2* (the enzyme encoded by this gene catalyses the conversion of UDP-Gal to produce galactinol, a precursor to Raf) [36] improved the cold resistance of rice by increasing the Raf content. In our study, no obvious difference in the expression of this gene was observed, but the raffinose synthase gene was upregulated in both S2 vs. S1 and S3 vs. S2. Thus, the key genes that may be responsible for promoting Raf accumulation and improving plant cold tolerance differ among species. The key gene may be the raffinose synthase gene in the primary rhizome bud of *P. kingianum*. Additionally, Suc was also produced during Raf metabolism (Fig. 5), possibly explaining the increase in Suc, although increased Suc catabolism occurred at the S3 stage.

### Interactions between carbohydrate and hormone catabolism

Several studies have reported the regulatory effect of hormones on carbohydrate metabolism during fruit development [37]. The present study also showed that interactions between carbohydrates and hormones occurred during bud dormancy release induced by chilling in *P. kingianum*.

Exogenous ethylene treatment of potato tubers can promote sugar (Suc, Glc and Fru) accumulation [38], which is similar to the effect of cold incubation [39]. Additionally, gene mutations related to ethylene signalling pathways caused changes in the sugar response [40]. Previous studies have indicated that crosslinks might exist among genes related to ethylene and sugar. Interestingly, strong interactions between ethylene-related genes (*SAM*, *ERF110*, *CTR1* and *EBF2*) and *SUS3/SPS* were observed in our network. This finding was consistent with previous results showing that exogenous ethylene treatment could increase *SPS* expression and the protein content [41]. In addition, GA_3_ can significantly enhance *SPS* expression during the pear ripening process [42] and *Paeonia lactiflora* dormancy release [43].

*BRI1*, a key gene in BR signal transduction, was present in our network and interacted with *PDH* and *STS. PDH* is a key gene involved in the aerobic oxidation of Glc, a finding that might support the previous conclusions that BR signal transduction is closely related to the sugar content and that *BRI1* regulates growth and development in response to sugars [44, 45]. Additionally, genes related to BR synthesis, such as *HYD1*, *FK.1*, *DWF4* and *SMT1,* were positively related to starch metabolism. Interestingly, *HYD1* was identified as a hub gene in the network (Fig. 6), strongly suggesting that BR played an important regulatory role in carbohydrate metabolism during bud dormancy release in *P. kingianum*. BR can stimulate sugar signalling and affect the sugar content [46, 47].

The interactions between IAA and carbohydrates were complex, and several related genes, such as *ARFs*, *TSA1, NIT4, YUCs, PINs* and *LAX3,* were observed and distributed among several links with carbohydrate catabolism. Studies on the relationship between carbohydrates and hormones have mainly focused on Suc and IAA. For example, the polar transport of IAA contributes to Suc absorption; in turn, Suc could induce IAA biosynthesis [48, 49]. This interaction might also exist during bud dormancy release in *P. kingianum*. Additionally, we observed other interesting interactions centred on *SIP1* and *STS,* which encode raffinose synthase and stachyose synthase, respectively. Several genes related to IAA, cytokinins, jasmonic acid and ethylene signal transduction interacted with the two genes, providing new insights to explore the signalling network.

### Proposed model of carbohydrate metabolism in response to cold treatment to regulate bud dormancy in *P. kingianum*

**Fig. 7:**
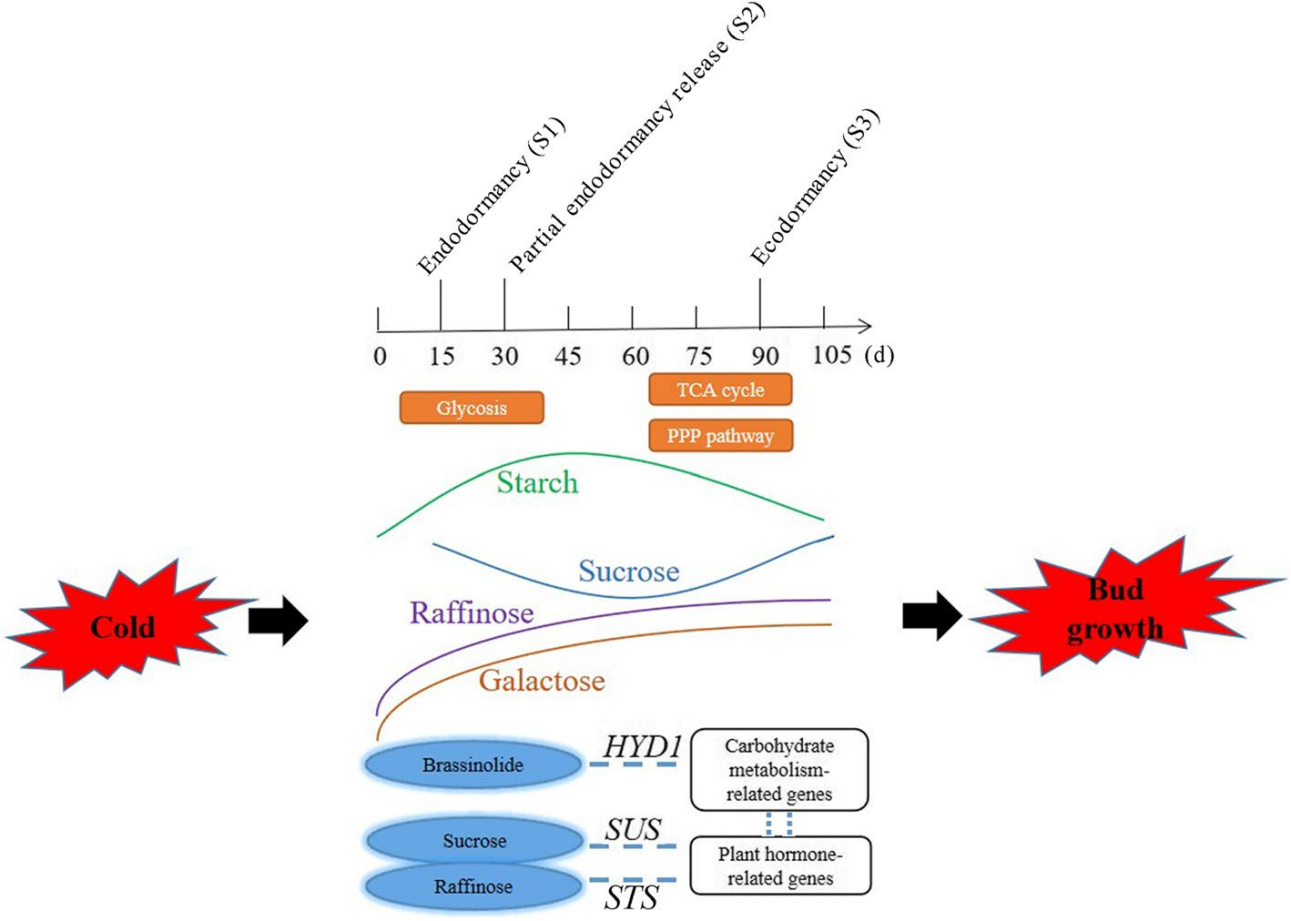
A proposed model of carbohydrate regulation response to chilling during dormancy release in *P. kingianum*

Based on the above results, a proposed model of carbohydrate regulation in response to cold temperature during rhizome bud dormancy in *P. kingianum* is provided (Fig. 7). The starch and Suc levels show opposing trends, which may be a critical signal of dormancy release in the *P. kingianum* rhizome bud. The levels of Gal and Raf increase with rhizome bud dormancy release, suggesting positive regulatory effects on dormancy release. At the initial stage of dormancy release in *P. kingianum*, anaerobic oxidation of the glycolysis pathway may be increased. After entering ecodormancy, the TCA cycle and PPP are possibly activated. *HYD1* is regarded as a hub gene interacting with numerous carbohydrate metabolism-related genes; additionally, crosslinks guided by *STS*/*SUS* between Raf/Suc and hormones also occur, suggesting that BR, Suc and Raf synergistically control the bud dormancy transition of *P. kingianum* under cold treatment. In this model, *HYD1, a* BR biosynthetic gene, plays a crucial role in response to cold treatment to regulate carbohydrate metabolism and bud dormancy. BR accumulation may be required to release bud dormancy in *P. kingianum* [2].

## Conclusions

This study defined carbohydrate changes in response to cold treatment during bud dormancy release in *P. kingianum.* In this process, starch and Suc showed opposite trends, Gal and Raf exhibited increases in both content and synthesis, and various changes also occurred in Glc, Cel, Man, Ara, Rha and Sta. Additionally, Glc metabolism was dominated by glycolysis in the early dormancy stage, and the PPP and TCA cycle were more active with dormancy gradually released. Interestingly, cross-links occurred between carbohydrates and hormones, and HYD1 was identified as a hub gene in the gene network during bud dormancy release in *P. kingianum*.

## Supplementary Information

**Additional file 1: Figure S1.** Morphogenesis process of *P. kingianum. *(A) Seed of *P. kingianum*. (B) Three days after the hypocotyl broke through the seed coat, the seed was cultivated for 20 days at 25°C. (C) The epicotyl begins to swell into a rhizome. (D-F) The rhizomes continue to swell, and the roots (D) and buds (F) form. (F) The rhizome bud ceases growth and enters endodormancy after the seed has been cultivated for 60 days at 25°C. (G) The rhizome bud grows into a seedling after dormancy is released by cold treatment at 4°C for 30 days followed by 15 days at 25°C. (H-I) The seedlings continue to grow for another 40-60 days after stage G.

**Additional file 2: Figure S2.** qRT–PCR results of four genes at different dormancy stages of *P. kingianum* rhizome buds. Proteins encoded by the *SUS3, SPS3, SIP1 *and* STS* genes are responsible for catalysing the conversion of Suc to UDPG and fructose, UDPG to Suc-6P, inositol galactoside to raffinose, and raffinose to stachyose, respectively.

**Additional file 3: Figure S3.** Analysis of the glycolysis pathway in S2 vs. S1 during rhizome bud dormancy release. Genes marked with red were upregulated, and genes marked with blue were downregulated.

**Additional file 4: Figure S4.** Analysis of the nucleotide sugar metabolism pathway in S2 vs. S1 during dormancy release. Genes marked with green were downregulated.

**Additional file 5: Figure S5.** Analysis of the nucleotide sugar metabolism pathway in S3 vs. S2 during dormancy release. Genes marked with red were upregulated.

**Additional file 6: Table S1.** Log_2_FC value of each gene related to 6 carbohydrate metabolism pathways.

**Additional file 7: Table S2.** Parameters generated by gene coexpression analysis.

## Data Availability

All the data used in this study are included in the article with its supplementary material. We have deposited our transcriptome data in the Sequence Read Archive (SRA) (http://www.ncbi.nlm.nih.gov/sra/), and the accession number for our submission is SRP151614.
