# Supplementary figures and images for: Carbohydrate regulation response to cold during rhizome bud dormancy release in Polygonatum kingianum
Source: BMC Plant Biol. 2022 Apr 1;22:163. doi: 10.1186/s12870-022-03558-0 (PMC8973533; doi:10.1186/s12870-022-03558-0)

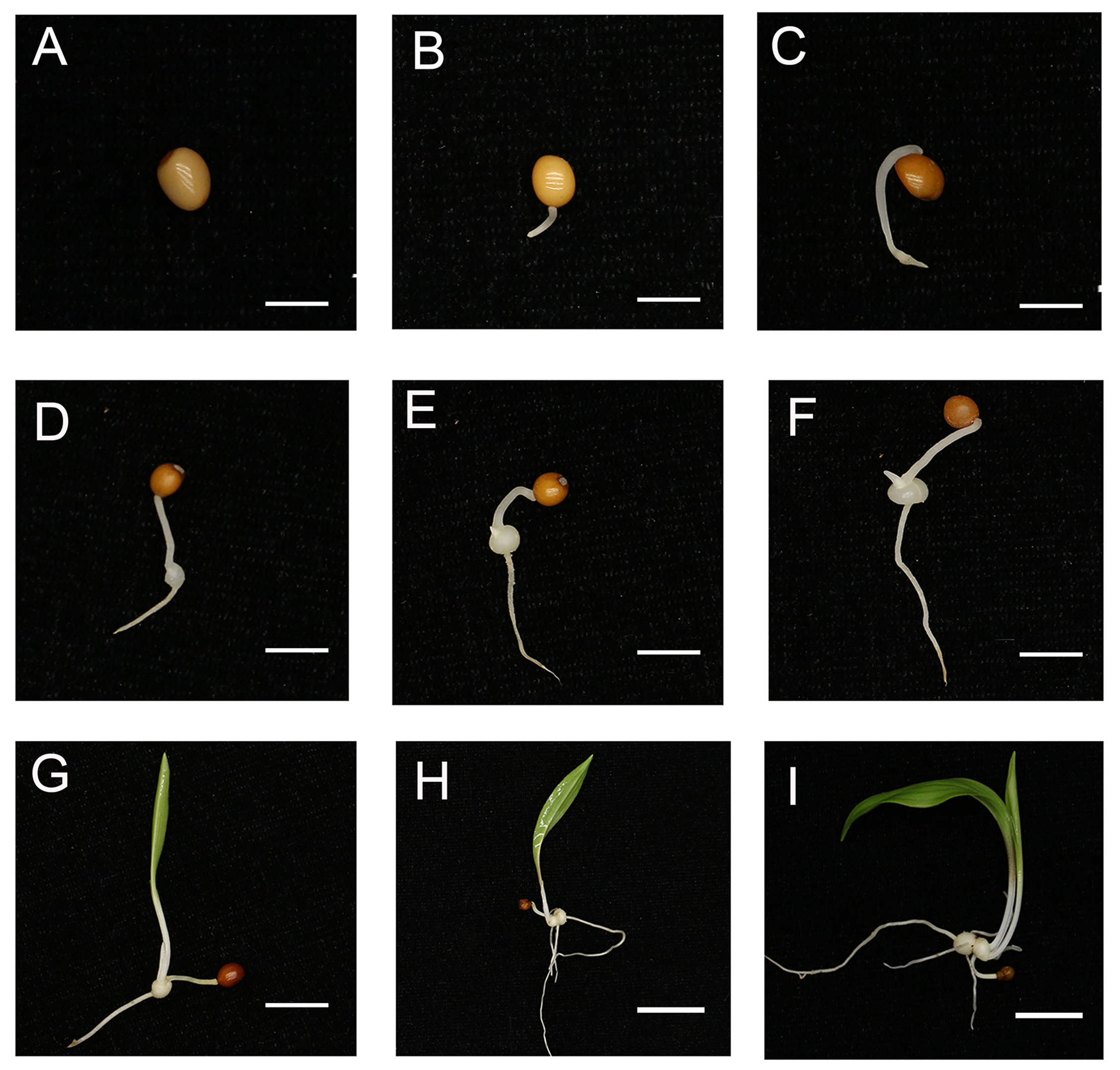

Supplement: Supplementary file 1 — Additional file 1: Figure S1. Morphogenesis process of P. kingianum. (A) Seed of P. kingianum. (B) Three days after the hypocotyl broke through the seed coat, the seed was cultivated for 20 days at 25°C. (C) The epicotyl begins to swell into a rhizome. (D-F) The rhizomes continue to swell, and the roots (D) and buds (F) form. (F) The rhizome bud ceases growth and enters endodormancy after the seed has been cultivated for 60 days at 25°C. (G) The rhizome bud grows into a seedling after dormancy is released by cold treatment at 4°C for 30 days followed by 15 days at 25°C. (H-I) The seedlings continue to grow for another 40-60 days after stage G. [file 12870_2022_3558_MOESM1_ESM.tif]

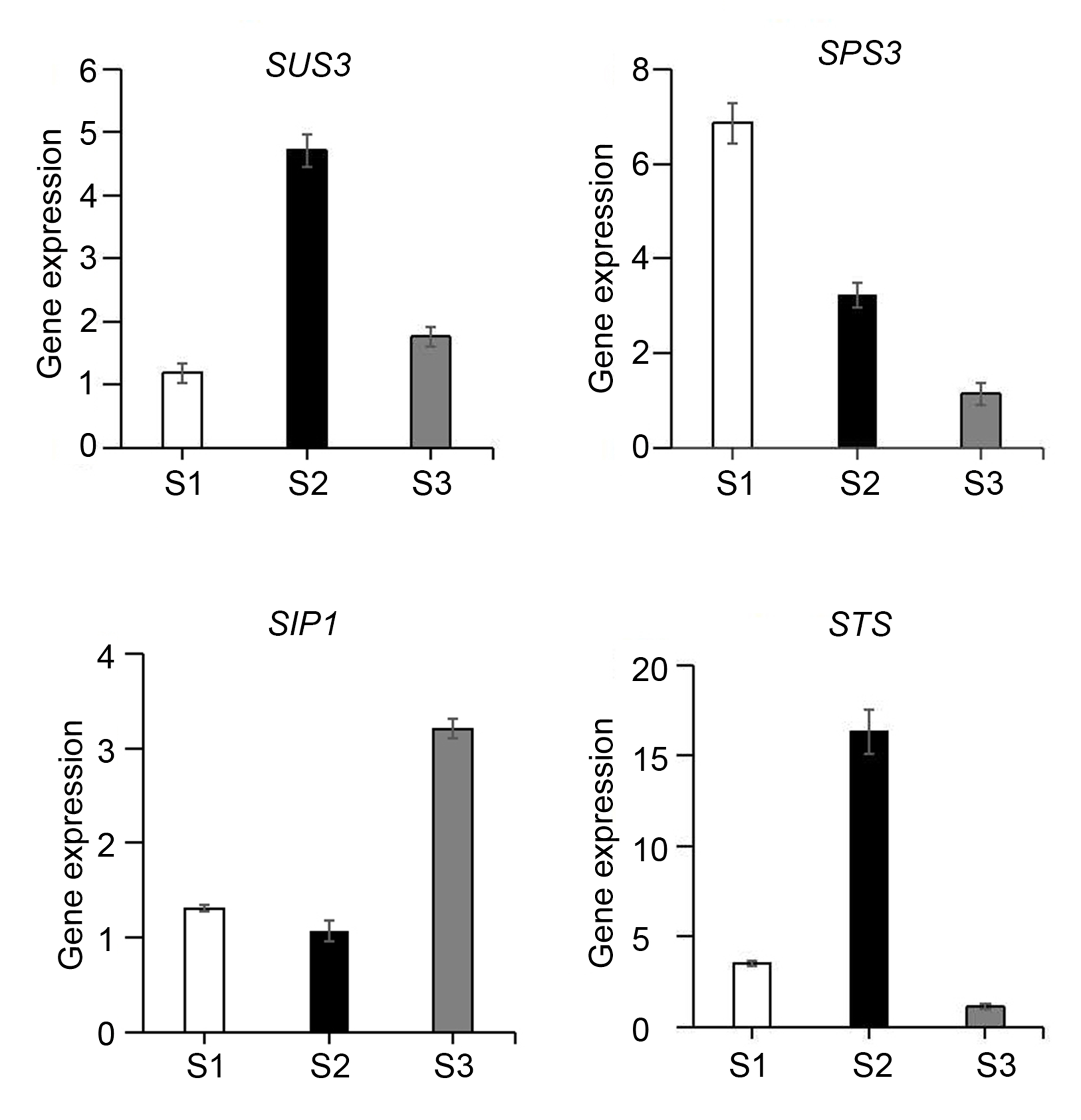

Supplement: Supplementary file 2 — Additional file 2: Figure S2. qRT–PCR results of four genes at different dormancy stages of P. kingianum rhizome buds. Proteins encoded by the SUS3, SPS3, SIP1 and STS genes are responsible for catalysing the conversion of Suc to UDPG and fructose, UDPG to Suc-6P, inositol galactoside to raffinose, and raffinose to stachyose, respectively. [file 12870_2022_3558_MOESM2_ESM.tif]

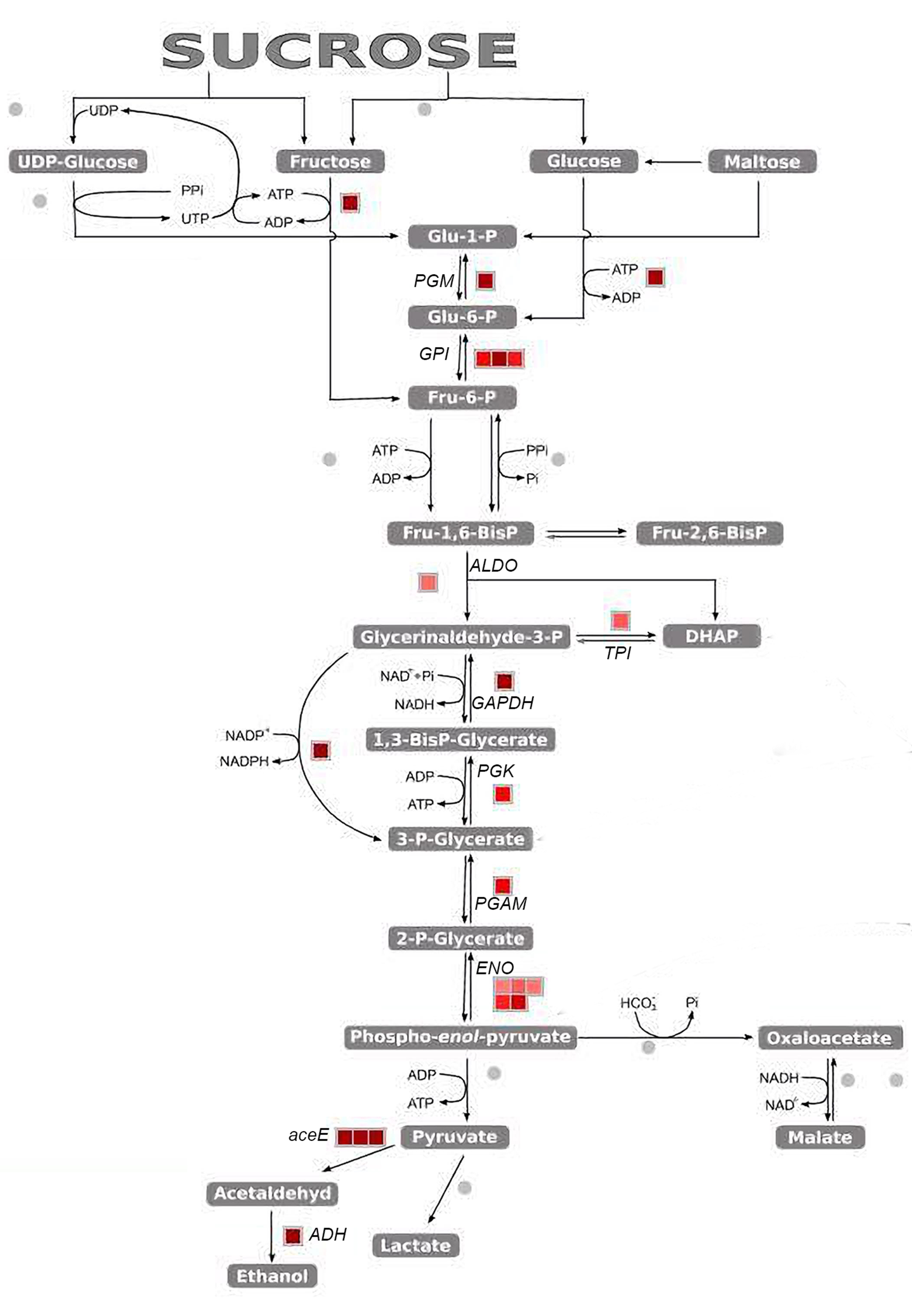

Supplement: Supplementary file 3 — Additional file 3: Figure S3. Analysis of the glycolysis pathway in S2 vs. S1 during rhizome bud dormancy release. Genes marked with red were upregulated, and genes marked with blue were downregulated. [file 12870_2022_3558_MOESM3_ESM.tif]

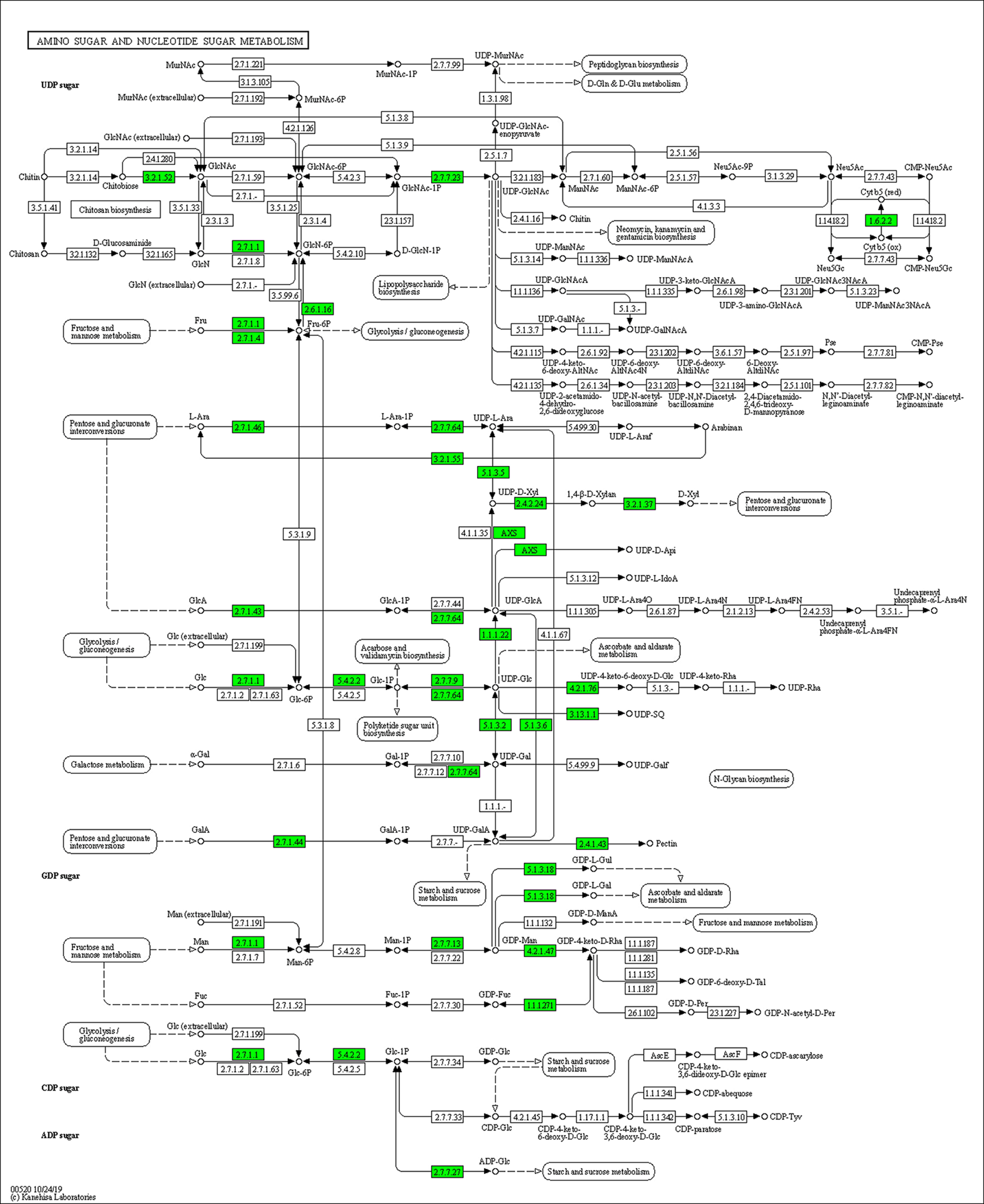

Supplement: Supplementary file 4 — Additional file 4: Figure S4. Analysis of the nucleotide sugar metabolism pathway in S2 vs. S1 during dormancy release. Genes marked with green were downregulated. [file 12870_2022_3558_MOESM4_ESM.tif]

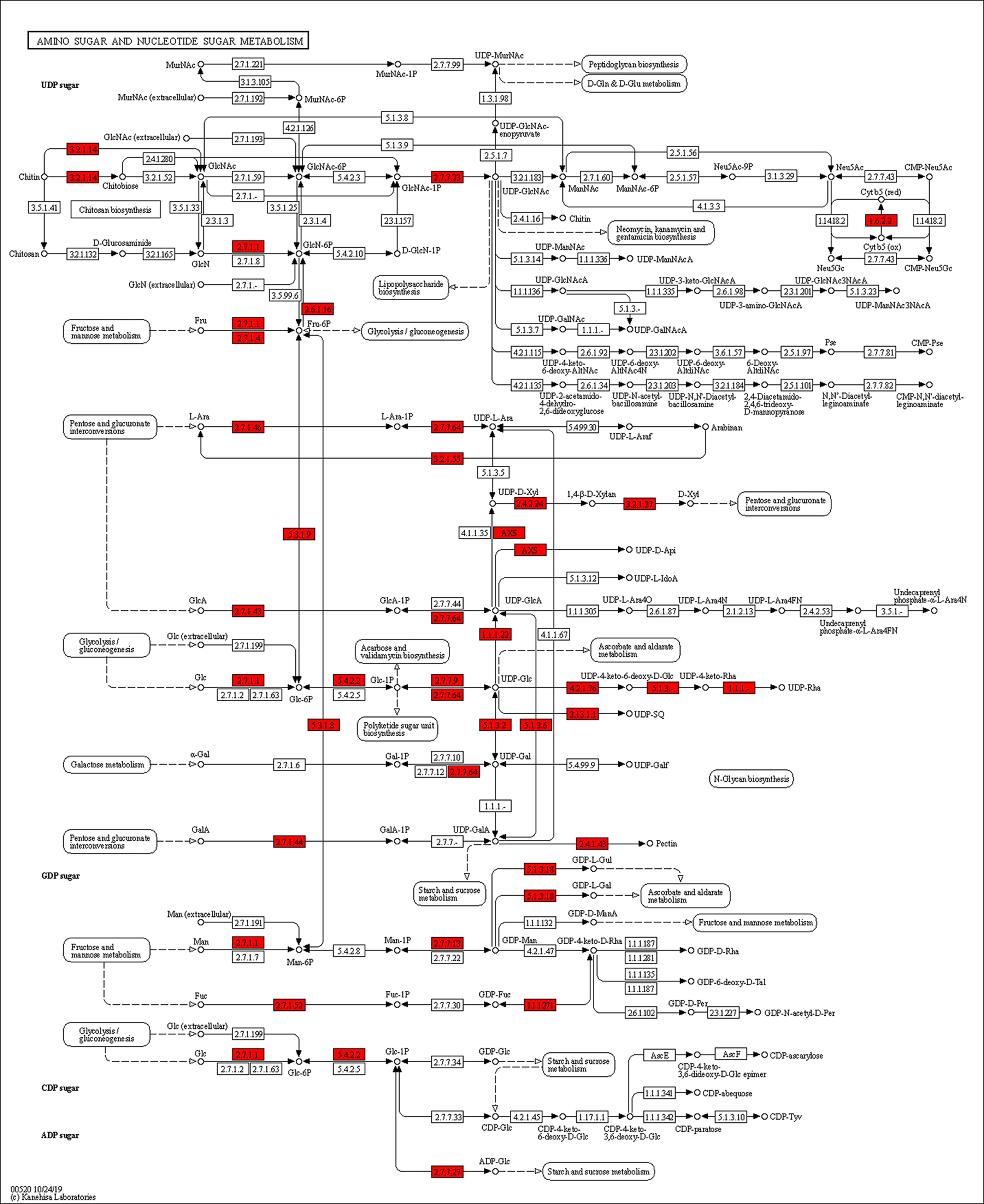

Supplement: Supplementary file 5 — Additional file 5: Figure S5. Analysis of the nucleotide sugar metabolism pathway in S3 vs. S2 during dormancy release. Genes marked with red were upregulated. [file 12870_2022_3558_MOESM5_ESM.tif]
